# A Case of Loss of Nearly Half of the Bones of the Skull, with Exposure and Sloughing of a Portion of the Brain

**Published:** 1873-10

**Authors:** Wm. W. Rutherford, H. Seaman

**Affiliations:** Harrisburg, Pa.; Millport, Chemung Co., N. Y.


### A Case of Loss of Nearly Half of the Bones of the Skull, with Exposure and Sloughing of a Portion of the Brain.

By Wm. W. Rutherford, M. D., of Harrisburg, Pa., and H. Seaman, M. D., of Millport, Chemung Co., N. Y.

On the morning of the 23d of July, about three o'clock, I was requested to visit Mr. Edward Thomas, at Highspire, a village on the Pennsylvania Canal, six miles east of Harrisburg, who was said to be seriously injured by his head striking against a canal bridge whilst asleep on the deck of his boat.

I reached Highspire about 4½ o'clock, and found Mr. T. in bed, his hair filled and matted with blood, his vest, shirt, upper part of his pantaloons and bed saturated with it, and a horrible looking rent in the scalp from the right superciliary ridge to the occipital bone. The wound was filled with coagulated blood, which stood up high above the level of the surrounding parts, and some blood still oozed from the wound. In a cloth, on a bench on the opposite side of the cabin, was rolled up a portion of the malar bone, some fragments of the os frontis, and the entire right parietal, detached from its fellow along the sagittal suture, and from the occipital along the lambdoidal suture, or perhaps taking some part of the occipital bone with it, together with the squamous portion of the temporal bone. It was as clear of soft parts as if it had been removed from the dead subject with scalpel and saw.

His pulse was small, moderately frequent, and rather feeble; skin rather below the natural temperature, but not much. Said he did not suffer much pain. His mind was perfectly undisturbed, quick and vigorous. I asked him if the sight of the right eye was impaired; he closed the left with his hand and said the vision of the right was perfect. He had no feeling of faintness, sickness of stomach, or any symptoms of concussion of the brain. The diminished force and volume, and increased frequency of the pulse were, I think, owing entirely to the loss of blood.

I suggested to Dr. Putt, who was in attendance with me, that it would be very difficult to dress the wound in the position in which Mr. Thomas was then lying. Mr. Thomas said he would sit up, and immediately got up and seated himself on a chair in the middle of the cabin floor.

We removed the hair an inch and a half or two inches from each side of the wound with scissors, and then shaved the scalp with a razor. I then examined the wound carefully with my finger, and found two loose pieces of bone about the superciliary ridge, which I removed. I then took a pocket-case spatula and commenced at the posterior angle of the wound, and removed a sufficiency of the coagulum to allow the edges of the scalp to be brought together by suture, then proceeded to remove some more and introduce another stitch, &c., until I had the wound in its whole extent very neatly brought together. In the clots which I removed I did not discover any discharged brain, nor did I get a sight of the membranes of the brain, for I was apprehensive if I removed the coagulated blood entirely that fresh hemorrhage would ensue. Indeed I concluded the less the brain was meddled with in that unprotected state the better.

The dressing occupied an hour or perhaps more, at the end of which he rose to his feet and removed his vest and shirt and put on a clean one; he then took of his pantaloons, and being handed a clean pair, poised himself on one foot and thrust the other into the leg of the pantaloons, changed feet, and thrust in the other leg, drew them up, buttoned and adjusted them with care, just as if nothing had happened to him, and walked over to his bed and laid himself down. He was not aware he had lost so much bone, or perhaps any, for it was concealed from him.

About 6½ o'clock I left him after applying a wet towel to his head, and at ten o'clock saw him again in company with two of our Harrisburg physicians, as the boat passed through the locks at this place.

Considerable reaction had occurred; his pulse was full, tolerably strong, and about 80; skin warm, mind clear but little pain, scarce any drowsiness, and his feelings he said quite comfortable.

If it were not for the fact that two physicians of this place, and Dr. Putt, of Highspire, have seen the patient, together with Dr. Seaman's letters, I should doubt the propriety of publishing it in a respectable medical journal, for really it is almost too marvelous for belief. Here is a man with nearly half of his skull torn away without any cerebral disturbance whatever, indeed without any symptoms to indicate the injury he has received except the torn scalp and the hemorrhage. Thus I conclude a hasty but truthful statement of the case as it came under my observation.

Since the accident, I have learned that it was produced by the end of one of the suspension rods which holds the string-pieces to the arch, the end of which projected below the timbers.

The boat was a very large one, used in carrying down coal, was returning empty, and floated very high, which accounts for the disaster.

---

MONTOURSVILLE, July 30, 1857.

DR. RUTHERFORD—

DEAR SIR: My brother-in-law, Edward Thomas, the boat captain whose head was so seriously injured by a bridge with so much loss of the bony structure, which was dressed by you on the morning of the 23d inst., near Harrisburg, requests me to write to you, and inform you that he is still living, and in full possession of all his mental faculties.

The dressing has not been removed from the wound, but he is apparently doing well. He sleeps comfortably during the night and occasionally during the day. His appetite for food is good, and he complains bitterly of the low diet to which he is subjected. Pulse ranges from 70 to 78, soft, and circulation equal. No preternatural heat of the skin. Complains of some dull pain in the head, helps himself up with ease, but when he starts up suddenly, as he sometimes does, from sleep, there will take place immediately considerable hemorrhage from the wound. On the whole, he is very comfortable, and hopes are beginning to be entertained by his friends of his ultimate recovery.

I have practiced medicine and surgery twenty-six years of my life, and had supposed that I had witnessed almost every form of human injury and suffering, but never before have I met any injury which would compare with this, and the patient so long survive after its infliction. For your gratification (as I presume you did not measure,) I will give you the actual measurement in a straight line across the concave surface of the piece of skull broken out, which now lies before me. You will recollect it was of an oval form, and I find it measures 6¾ inches in its longest diameter, and 5¾ inches in its shortest diameter.

With such a loss of the bony covering of the brain and the violence of the blow necessary to remove it, the great wonder is that the patient is still alive and comfortable, on this, the eighth day after the accident.

Yours Truly,

H. SEAMAN.

---

MONTOURSVILLE, August 5, 1857.

DR. WM. W. RUTHERFORD—

DEAR SIR: Your very obliging letter of the 2d inst. is received, and it affords me much pleasure to comply with your request to keep you informed of Edward Thomas' condition

This is the thirteenth day since he received the injury, and strange as it may appear, he is evidently doing well. During the first ten days succeeding the wound, there was considerable and frequent returns of hemorrhage from it, which would occur on almost every effort to sit up or even turn over in bed, but was readily arrested, in most instances, by the more frequent application of ice water. Since suppuration has commenced the bleeding has ceased.

I removed your dressing on the eighth day, and found the sutures all sloughed out, and no union of the wound by the first intention. The edges of the wound were widely parted, the scalp hanging in a fold over the ear, leaving a portion of the surface of the brain the length of the wound and one and one-half inch wide exposed to view. I have since. and with much difficulty, shaved off the entire scalp and brought the edges of the wound nearly together by adhesive straps, supporting them by the application of a bandage to the entire head. The tightness of these dressings was made to depend on the feelings of the patient. Suppuration is gradually going on, and granulations forming over the surface of the dura mater. All his symptoms at present are favorable. Intellect perfect, appetite good, pulse varying from 76 to 90 in a minute, tongue clean, skin nearly natural, strength holds out well, sits up occasionally from one to two hours at a time, and his friends are beginning to entertain a hope of his ultimate recovery. The cold wet cloths are still applied, as they have been faithfully from the first, to the persevering application of which I think he owes his life and the comparatively comfortable condition he now enjoys.

You will please accept the thanks and the gratitude of the patient and his friends for your skillful and persevering effort to save the life of this young man in one of the most hopeless conditions ever falling under the notice of the medical profession. I shall be much obliged, not only to you, but Dr. Butler also for a copy of the number of the *Reporter*, containing a notice of this case. I will endeavor to keep you posted in reference to its progress.

I remain yours truly.

H. SEAMAN.

---

MONTOURSVILLE, August 8, 1857.

DR. WM. W. RUTHERFORD—

DEAR SIR: Again I write to inform you of Edward Thomas' condition. Since I wrote you last he has been improving rapidly. I have just finished dressing the wound, and find the floating scalp firmly attached to the dura mater in every part, and covered by it, except the exposed portion, a strip three-fourths of an inch wide by six inches long, and this is entirely covered by strong and healthy granulations. I have continued to dress the wound with long adhesive straps, keeping it clean by the use of a sponge and warm water.

He does not complain of as much pain in his head and ears as formerly, and sits up in a chair two or three hours per day. His appetite is good, rests well at night, and has been walking about the house this afternoon without much apparent fatigue. At present he is recovering very fast, and if no unfavorable change should take place, he will soon be quite well.

In a former statement which I made to you in reference to the amount of skull bone broken out, I committed an error by not having compared the portion broken out with that left. I there said "nearly one-third of the entire skull is broken out," but should have come nearer the truth, had I said nearly one-half instead of saying one-third. I had forgotten to mention above that his intellect remains undisturbed, and that considerable of the lower part of the right front lobe of the brain was so injured that it has sloughed away.

This case presents considerations for the physiologist and phrenologist, some of whom may jump to the conclusion, that men, in this fast age, do not require such cumbrous bony structures, filled with so much chaff called brains, as many of us carry on our shoulders.

I remain yours, &c.,

H. SEAMAN.

